# User Intentions to Use ChatGPT for Self-Diagnosis and Health-Related Purposes: Cross-sectional Survey Study

**DOI:** 10.2196/47564

**Published:** 2023-05-17

**Authors:** Yeganeh Shahsavar, Avishek Choudhury

**Affiliations:** 1 Industrial and Management Systems Engineering Benjamin M Statler College of Engineering and Mineral Resources West Virginia University Morgantown, WV United States

**Keywords:** human factors, behavioral intention, chatbots, health care, integrated diagnostics, use, ChatGPT, artificial intelligence, users, self-diagnosis, decision-making, integration, willingness, policy

## Abstract

**Background:**

With the rapid advancement of artificial intelligence (AI) technologies, AI-powered chatbots, such as Chat Generative Pretrained Transformer (ChatGPT), have emerged as potential tools for various applications, including health care. However, ChatGPT is not specifically designed for health care purposes, and its use for self-diagnosis raises concerns regarding its adoption’s potential risks and benefits. Users are increasingly inclined to use ChatGPT for self-diagnosis, necessitating a deeper understanding of the factors driving this trend.

**Objective:**

This study aims to investigate the factors influencing users’ perception of decision-making processes and intentions to use ChatGPT for self-diagnosis and to explore the implications of these findings for the safe and effective integration of AI chatbots in health care.

**Methods:**

A cross-sectional survey design was used, and data were collected from 607 participants. The relationships between performance expectancy, risk-reward appraisal, decision-making, and intention to use ChatGPT for self-diagnosis were analyzed using partial least squares structural equation modeling (PLS-SEM).

**Results:**

Most respondents were willing to use ChatGPT for self-diagnosis (n=476, 78.4%). The model demonstrated satisfactory explanatory power, accounting for 52.4% of the variance in decision-making and 38.1% in the intent to use ChatGPT for self-diagnosis. The results supported all 3 hypotheses: The higher performance expectancy of ChatGPT (β=.547, 95% CI 0.474-0.620) and positive risk-reward appraisals (β=.245, 95% CI 0.161-0.325) were positively associated with the improved perception of decision-making outcomes among users, and enhanced perception of decision-making processes involving ChatGPT positively impacted users’ intentions to use the technology for self-diagnosis (β=.565, 95% CI 0.498-0.628).

**Conclusions:**

Our research investigated factors influencing users’ intentions to use ChatGPT for self-diagnosis and health-related purposes. Even though the technology is not specifically designed for health care, people are inclined to use ChatGPT in health care contexts. Instead of solely focusing on discouraging its use for health care purposes, we advocate for improving the technology and adapting it for suitable health care applications. Our study highlights the importance of collaboration among AI developers, health care providers, and policy makers in ensuring AI chatbots’ safe and responsible use in health care. By understanding users’ expectations and decision-making processes, we can develop AI chatbots, such as ChatGPT, that are tailored to human needs, providing reliable and verified health information sources. This approach not only enhances health care accessibility but also improves health literacy and awareness. As the field of AI chatbots in health care continues to evolve, future research should explore the long-term effects of using AI chatbots for self-diagnosis and investigate their potential integration with other digital health interventions to optimize patient care and outcomes. In doing so, we can ensure that AI chatbots, including ChatGPT, are designed and implemented to safeguard users’ well-being and support positive health outcomes in health care settings.

## Introduction

### Background

The digital age has witnessed an unprecedented surge in technological innovation, shaping the essence of human-computer interaction. As the world progresses toward a future encompassing artificial intelligence (AI), advanced conversational AI models, such as Chat Generative Pretrained Transformer (ChatGPT), a cutting-edge conversational AI model by OpenAI, have come to the forefront of academic discussion. This paradigm-shifting technology has revolutionized our interactions with machines and introduced profound implications across multiple disciplines. By harnessing the power of machine learning, ChatGPT transcends the limitations of traditional chatbots, yielding increasingly humanlike conversational capabilities. The technology has demonstrated remarkable capabilities, such as understanding context, generating coherent text, and adapting to various natural language processing (NLP) tasks, including but not limited to language translation, answering of questions, and text generation [1]. The success of these models can be attributed to their scale, as they have been trained on vast amounts of data from diverse sources, such as books, papers, and websites [2]. By leveraging these extensive training data, ChatGPT has learned patterns, syntax, and semantics, enabling it to generate humanlike responses, making it a valuable tool in many applications and industries [3].

The literature has demonstrated the potential of AI-based chatbots, such as ChatGPT, to revolutionize patient care and service delivery [4-6]. Numerous recent studies have underscored the potential of ChatGPT in the health care sector [7]. For instance, 1 investigation delved into the capabilities of ChatGPT across a range of clinical situations, discovering its potential to enhance patient communication and engagement within health care contexts. The study found that ChatGPT effectively delivers information and support to patients in various scenarios, such as mental health assessments, counseling, medication management, and patient education [8]. A recent review examined the advantages of ChatGPT and other large language models in augmenting medical education, streamlining clinical decision-making, and promoting better patient outcomes [9].

However, ChatGPT is not specifically trained in medical literature. It is crucial to understand the intended purpose of ChatGPT and acknowledge its negative consequences if used otherwise. The use of ChatGPT in health care has raised concerns about the accuracy and reliability of the information provided, patient privacy and data security, prejudice, responsibility, and the ethical ramifications of using such potent language models. A study also emphasized the importance of using strong cybersecurity measures to protect patient data and privacy when using ChatGPT in health care settings [10].

Although ChatGPT has proven to be a remarkable technological achievement, its application in self-diagnosis poses significant risks that must be noted. We all have used the internet for self-diagnosis. Depending on the user’s health literacy, the source’s validity, and the accuracy of information interpretation, web-based self-diagnosis has resulted in positive and negative consequences. Just like most consumer-facing screen-based technologies, ChatGPT has the potential for misinterpretation and misuse, necessitating a careful approach to implementation. This is important because misuse (using it for tasks it is not designed for) can affect user trust, resulting in the underuse of the technology [11]. The convenience and accessibility of ChatGPT have made it appealing for self-diagnosis purposes, much like the broader internet. With an internet connection, ChatGPT can be easily accessed anytime and anywhere, allowing individuals to seek diagnostic information without needing a physical appointment or incurring medical costs. This ease of access can be incredibly enticing for those with limited access to health care services or who face financial constraints. Another factor contributing to the appeal of ChatGPT for self-diagnosis is the sense of anonymity and privacy it provides. Discussing sensitive health issues can be uncomfortable or embarrassing, leading individuals to prefer the discretion offered by an AI-based chatbot over face-to-face consultations with health care professionals. ChatGPT delivers prompt responses, providing instant feedback to users’ inquiries. This immediacy can attract those seeking quick answers or reassurance about their health concerns. Additionally, as ChatGPT is an AI-driven language model built on vast amounts of data and because of its promising performance in several fields, users may perceive it as a knowledgeable and reliable source of information. This perceived expertise can create a false sense of confidence in the diagnostic suggestions provided by ChatGPT, despite its inherent limitations.

We must understand that anyone with a computer and an internet connection can use ChatGPT. Individuals with minimal to no health and technology literacy may not realize the limitations of ChatGPT and its intended use. User character, the intricacy of medical information, and the unique nature of individual patient cases underscore the potential for misinterpretation. Inaccurate or incomplete information provided by ChatGPT may result in misguided self-diagnosis or exacerbation of existing conditions. From a cognitive human factor standpoint, the misalignment between AI-generated information and users’ mental models can lead to erroneous decision-making and unfavorable health outcomes. The possibility of ChatGPT being misused for self-diagnosis is a significant concern. To counteract this, accessing the potential misuse of ChatGPT from a human factor standpoint is essential.

Addressing the concerns associated with AI chatbots in health care, this study aims to (1) explore users’ intentions to use ChatGPT for self-diagnosis and (2) gain a deeper understanding of the factors influencing their decision-making processes. By providing novel insights into the implications of AI chatbot adoption in health care settings, we intend to inform the development of guidelines, policies, and interventions that promote the responsible and effective use of AI technologies in health care. The originality of this study stems from its focus on users’ decision-making processes and intentions when using AI chatbots for self-diagnosis, an area of research that remains relatively unexplored in the context of health care applications.

**Figure 1:**
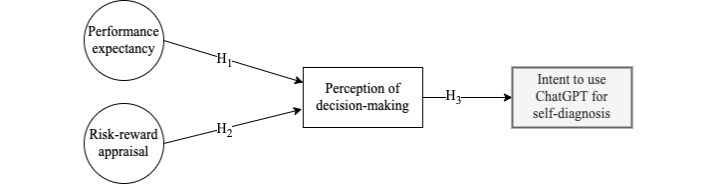
Conceptual framework illustrating the effect of performance expectancy and risk-reward appraisal on the perception of decision-making (directly) and intent to use ChatGPT for self-diagnosis (indirectly). ChatGPT: Chat Generative Pretrained Transformer; H: hypothesis.

As illustrated in Figure 1, our investigation examines the effects of the perceived effectiveness and risk-benefit appraisal of ChatGPT on decision-making and the subsequent impact on users’ intent to use ChatGPT for self-diagnosis. This examination is crucial due to AI technologies’ rapid growth and adoption across various aspects of daily life, including health care and self-diagnosis [12]. Gaining a comprehensive understanding of the factors driving user acceptance, trust, and adoption of AI technologies is essential to ensure their responsible and efficient use. Additionally, scrutinizing the potential implications of these effects is critical for informing guidelines and policies surrounding AI technologies like ChatGPT for self-diagnosis [13].

By pinpointing the factors contributing to users’ decision-making processes and intentions to use ChatGPT, regulators and health care professionals can develop informed policies and recommendations to ensure AI’s safe and ethical deployment in health care [14]. Adopting this approach will help mitigate potential misuse or overreliance on such technologies for self-diagnosis, which could result in misdiagnosis or delayed treatment.

### Theory and Hypotheses Development

The conceptual framework (see Figure 1) explored in this study was inspired by the unified theory of acceptance and use of technology (UTAUT). UTAUT is an established theoretical framework extensively used for comprehending and predicting individuals’ technology adoption and usage [15]. The UTAUT framework posits 4 factors influencing an individual’s behavioral intention to use a given technology: performance expectancy, effort expectancy, social influence, and facilitating conditions. In this research, we only retrieved performance expectancy from UTUAT and added risk-benefit considerations and decision-making as factors affecting the intent to use ChatGPT.

### Operational Definitions

In this study, performance expectancy is operationally defined as the extent to which an individual anticipates that using ChatGPT will augment their capacity to accomplish tasks, attain objectives, and alleviate workload proficiently and efficiently. This latent construct encapsulates the user’s perceptions regarding the advantages, effectiveness, and overall satisfaction derived from their interaction with ChatGPT.

The construct of decision-making was operationally defined as the extent to which an individual perceives ChatGPT as a valuable tool for assisting them in making informed, timely, and effective choices by providing relevant recommendations and insights. This latent construct encompasses the user’s belief in ChatGPT’s ability to contribute positively to their decision-making process and their willingness to act on the recommendations generated by the technology.

Similarly, the risk-reward-appraisal construct can be operationally defined as the extent to which an individual perceives the advantages of using ChatGPT as surpassing any potential adverse consequences or risks associated with its use. This latent construct captures the user’s evaluation of the trade-offs between the positive outcomes derived from ChatGPT and the potential hazards or drawbacks that may arise from its implementation.

### Hypotheses

The following are the hypotheses tested in this study.

#### Hypothesis 1

The higher performance expectancy of ChatGPT is positively associated with improved user decision-making outcomes. Hypothesis 1 (H1) is grounded in established theories, such as the technology acceptance model (TAM) and UTAUT [15,16]. These theories posit that performance expectancy is critical to technology acceptance and usage intentions. Trust in a technology, which is positively associated with performance expectancy [17], further supports the notion that when users have higher trust in ChatGPT’s ability to perform effectively, they are more likely to rely on its recommendations, thus positively influencing their decision-making processes [11]. Cognitive fit theory complements this relationship by suggesting that the alignment between an individual’s cognitive processes and the representation of information by technology influences the effectiveness of problem solving and decision-making [18]. As users perceive ChatGPT as an effective tool that aligns with their cognitive processes and expectations, they are more inclined to incorporate its recommendations into their decision-making, leading to improved outcomes.

Although this is the first study to explore the impact of the perceived effectiveness of ChatGPT on decision-making, H1 can be justified by drawing on several studies that have explored the relationship between performance expectancy and technology acceptance, usage, or decision-making in other domains. For instance, Al-Emran et al [19] conducted a systematic review investigating the impact of performance expectancy on mobile learning adoption, revealing its positive effect on learners’ intentions to use mobile technologies for educational purposes. This study further emphasizes the importance of performance expectancy in shaping users’ engagement with technology and their inclination to rely on it for decision-making. Lee and Kozar [20] explored the relationship between website quality, user satisfaction, and decision-making in e-business contexts. Their findings demonstrated that when users perceive a website as effective and efficient, they are more likely to trust its recommendations and make decisions based on the provided information. This study underscores the significance of performance expectancy in users’ trust and decision-making behaviors. Another study proposed that managers’ perceived usefulness and ease of use of AI are significant predictors of their intention to use AI for decision-making in organizations [21]. In a study by Alaiad and Zhou [22], the determinants of health care professionals’ intention to adopt AI-based clinical decision support systems were examined, focusing on factors such as performance expectancy, effort expectancy, social influence, and facilitating conditions. The researchers proposed an extended TAM tailored for health care, addressing the distinct requirements and challenges of the health care domain. The study offered insights into the factors that affect health care professionals’ decision-making processes and their intent to use AI technologies in their practice [22].

#### Hypothesis 2

A positive risk-reward appraisal of ChatGPT is associated with enhanced user decision-making outcomes. Hypothesis 2 (H2) is based on established psychological and decision-making theories, such as prospect theory and protection motivation theory (PMT), as well as the concept of trust in technology [17,23,24].

Prospect theory posits that individuals evaluate potential gains and losses during decision-making processes and that their choices are influenced by the perceived risks and rewards associated with each alternative [23]. In the context of ChatGPT, users who perceive the benefits of using AI technology to outweigh the potential risks are more inclined to rely on it for decision-making purposes. PMT suggests that individuals’ intentions to engage in protective behaviors are influenced by their perceived severity of a threat, perceived vulnerability, response efficacy, and self-efficacy [24]. Applying PMT to ChatGPT implies that if users believe the benefits of using the technology (response efficacy) surpass the potential risks (perceived severity and vulnerability), they are more likely to integrate ChatGPT’s recommendations into their decision-making processes. Moreover, trust in technology has been identified as a crucial factor influencing technology adoption and usage [17]. When users perceive a favorable risk-reward balance, they are more likely to trust ChatGPT and subsequently rely on its recommendations for decision-making.

In technology adoption, risk perception can significantly affect decision-making. Although there may not be studies directly examining the relationship between risk-reward appraisal and decision-making in the context of ChatGPT, several studies have explored the impact of risk perception and trust in technology on decision-making and technology adoption in other domains. For instance, a study examined the interplay between trust, perceived risk, and TAM in the context of consumer acceptance of electronic commerce (e-commerce) [25]. The findings revealed that trust and perceived risk significantly influence users’ behavioral intentions. Users who perceived a favorable risk-reward balance were more inclined to engage with e-commerce platforms [25]. This suggests that a positive risk-reward appraisal could also influence decision-making processes involving ChatGPT. Another study developed a trust-based consumer decision-making model in e-commerce, emphasizing the importance of perceived risk and trust in users’ decision-making processes [26]. The study demonstrated that users who perceive a positive risk-reward balance when using e-commerce platforms are more likely to base their decisions on the information provided, further supporting the notion that risk-reward appraisal plays a crucial role in decision-making outcomes [26]. Lastly, a study explored the role of trust and risk perception in mobile commerce adoption. Their findings indicated that users who perceive a favorable risk-reward balance are likelier to adopt mobile commerce technologies and rely on them for decision-making [27]. This study highlights the significance of risk-reward appraisal in technology adoption and decision-making.

#### Hypothesis 3

A positive perception of ChatGPT’s role in enhancing decision-making processes is associated with an increased intention among users to use the technology for self-diagnosis. Hypothesis 3 (H3) can be substantiated by drawing upon well-established theories, such as TAM, UTAUT, and research on trust in technology [15-17]. TAM posits that users’ intention to adopt a technology is influenced by their perceptions of its usefulness and ease of use [16]. Consequently, if users view ChatGPT as a valuable decision-making aid that is user friendly, they are more likely to intend to use it for self-diagnosis purposes. TAM also asserts that users’ actual system usage is impacted by their behavioral intention, suggesting that positive decision-making experiences with ChatGPT could lead to increased use for self-diagnosis. According to UTAUT, when users experience effective decision-making processes that involve ChatGPT (technology), their performance expectancy (the extent to which they believe the technology will assist them in achieving their goals) may rise, thereby fostering a greater intent to use ChatGPT for self-diagnosis.

## Methods

### Ethical Considerations

The study, classified as a flex protocol type, received approval from the Institutional Review Board (IRB) of West Virginia University (IRB protocol number 2302725983). Informed consent was obtained from participants. The data gathered through the online survey were securely stored on Centiment’s platform and remained accessible exclusively to the research team.

### Survey Instruments

Table 1 lists the survey questions used in the study. We adapted questions from UTAUT to form the latent construct *performance expectancy*. The construct was established by aggregating questions related to 4 statements:

- Statement 1: “ChatGPT can help me achieve my goals.” This item gauges the user’s conviction regarding ChatGPT’s capability to facilitate the attainment of their desired objectives within the context of their tasks.
- Statement 2: “ChatGPT can reduce my workload.” This item appraises the user’s perception of ChatGPT’s potential to mitigate the burden of task completion by streamlining processes and increasing efficiency.
- Statement 3: “I was successful in achieving what I wanted to accomplish with ChatGPT.” This item measures the user’s perception of the degree to which their interaction with ChatGPT has led to the realization of intended outcomes, reflecting the efficacy of the technology in practical applications.
- Statement 4: “I am satisfied with ChatGPT.” This item examines the user’s overall contentment with the performance of ChatGPT, capturing their appraisal of its utility and effectiveness in addressing their needs and expectations.

In addition, 2 statements were developed to form the latent construct *decision-making*:

- Statement 1: “ChatGPT helps me make informed and timely decisions.” This item evaluates the user’s perception of ChatGPT’s capacity to provide pertinent information, insights, and guidance, which in turn facilitates well-informed and timely decision-making processes.
- Statement 2: “I am willing to make decisions based on the recommendations provided by ChatGPT.” This item measures the user’s trust in the recommendations offered by ChatGPT and their readiness to incorporate those suggestions into their decision-making processes.

**Table 1:** Statements used in the survey.

Factor	Questions
Performance expectancy (PE)	To what extent do you agree with the following: ChatGPT^a^ can help me achieve my goals (PE1). To what extent do you agree with the following: ChatGPT can reduce my workload (PE2). To what extent do you agree with the following: I was successful in achieving what I wanted to accomplish with ChatGPT (PE3). To what extent do you agree with the following: I am satisfied with ChatGPT (PE4).
Perception of decision-making (DM)	To what extent do you agree with the following: ChatGPT helps me make informed and timely decisions (DM1). To what extent do you agree with the following: I am willing to make decisions based on the recommendations provided by ChatGPT (DM2).
Risk-reward appraisal (RRA)	To what extent do you agree with the following: The benefits of using ChatGPT outweigh any potential risks (RRA).
Intent to use (IU)	To what extent do you agree with the following: I am willing to use ChatGPT for self-diagnosis purposes (IU).

^a^ChatGPT: Chat Generative Pretrained Transformer.

Furthermore, 1 statement measured the extent to which an individual perceives the advantages of using ChatGPT as surpassing any potential adverse consequences or risks associated with its use: “The benefits of using ChatGPT outweigh any potential risks.”

Lastly, users’ willingness to use ChatGPT for self-diagnosis was captured using 1 statement: “I am willing to use ChatGPT for self-diagnosis purposes.”

All the items were assessed using a 4-point Likert scale, allowing participants to indicate their level of agreement with each statement, ranging from “strongly disagree” to “strongly agree.”

Note that we used a forced Likert scale in this study. By precluding the inclusion of a neutral or midpoint option, a forced Likert scale necessitates respondents to articulate a definitive opinion or predilection, thereby generating data that are more incisive and unequivocal [28]. This approach proves particularly advantageous in scenarios where the research is intended to ascertain well-defined attitudes or perceptions from participants. Forced scales have been demonstrated to mitigate the acquiescence bias, a phenomenon wherein respondents are predisposed to concur with statements regardless of their content [29]. Eliminating a neutral option encourages participants to critically contemplate their responses, yielding more accurate data [28]. Furthermore, forced scales engender more reliable outcomes when assessing relatively polarized or fervently held attitudes [30]. By obliging participants to select between affirmative and negative response options, a forced scale can offer more lucid insights into the direction and intensity of their attitudes.

### Data Collection

The data collection for this study took place in February 2023, using an online survey administered through Centiment, a reputable service provider for survey deployment and data gathering [31]. By leveraging Centiment’s capabilities, the research team efficiently disseminated the survey. It ensured the participation of a diverse sample of respondents, specifically recruiting individuals who used ChatGPT at least once per month. Centiment’s robust platform facilitated the research team in designing and distributing the survey, while implementing various quality control measures and preventing duplicate responses. This approach bolstered the data’s reliability and validity. Furthermore, the online survey format allowed participants to complete it at their discretion, contributing to an increased response rate and enhanced sample diversity.

Upon obtaining informed consent from participants, they were directed to the survey, which contained questions designed to measure the constructs under investigation. The survey used forced 4-point Likert scale questions to elicit decisive responses from participants, thus reducing potential biases. Additionally, the survey incorporated a checking question to verify that respondents thoroughly read all questions before providing their answers, further ensuring data quality. Upon completing the data collection process, the team meticulously reviewed and processed the data to ascertain their quality and accuracy before advancing to subsequent data analysis.

### Data Analysis

The data analysis for this study consisted of 2 primary stages: descriptive statistics and partial least squares structural equation modeling (PLS-SEM). Descriptive statistics were calculated for all survey questions to provide an overview of the responses’ central tendency, dispersion, and distribution. These statistics offered initial insights into the participants’ attitudes and perceptions regarding the constructs under investigation.

Following the descriptive analysis, the research team used PLS-SEM to examine the relationships between the latent constructs. PLS-SEM is a powerful multivariate analysis technique that allows researchers to estimate complex cause-effect relationships between latent constructs and their indicators [32]. This method was chosen for its ability to handle small- to medium-size samples and suitability for exploratory research [33]. The PLS-SEM analysis in our study was conducted in 2 stages: the assessment of the measurement model and the evaluation of the structural model. We assessed the measurement model for reliability and validity by focusing on 4 aspects: indicator reliability, internal consistency reliability, convergent validity, and discriminant validity. Indicator reliability was examined by analyzing the factor loadings of each indicator, with loadings greater than 0.5 considered satisfactory. We evaluated internal consistency reliability using composite reliability (rhoC), and values above 0.7 were deemed acceptable [34]. Convergent validity was assessed by examining the average variance extracted (AVE), and values above 0.5 indicated an adequate convergent validity [34,35]. In addition to these assessments, we also evaluated the reliability of the constructs in our research model using the average interitem correlation (rhoA). Both rhoC and rhoA are measures of internal consistency that help determine how closely related the survey questions are within each construct. A value of 0.7 or higher for both rhoC and rhoA is generally considered to indicate satisfactory reliability.

After confirming the measurement model’s adequacy, we evaluated the structural model to test our hypotheses. This analysis included assessing the path coefficients, significance levels, and determination coefficients (*R*^2^) for each endogenous latent construct.

## Results

### Participant Details

A total of 607 individuals participated in the study, providing comprehensive responses to the questionnaire. Table 2 shows the descriptive statistics of the study variables. Most of the respondents used ChatGPT for information search and entertainment purposes. Others used the technology to solve problems and conduct health-related searches. Most respondents were willing to use ChatGPT for self-diagnosis (n=476, 78.4%). Most of the respondents were also familiar (to a certain degree) with the technology of ChatGPT and perceived the technology to be persuasive. Most respondents had a bachelor’s degree, a high school diploma, or a master’s degree.

**Figure 2:**
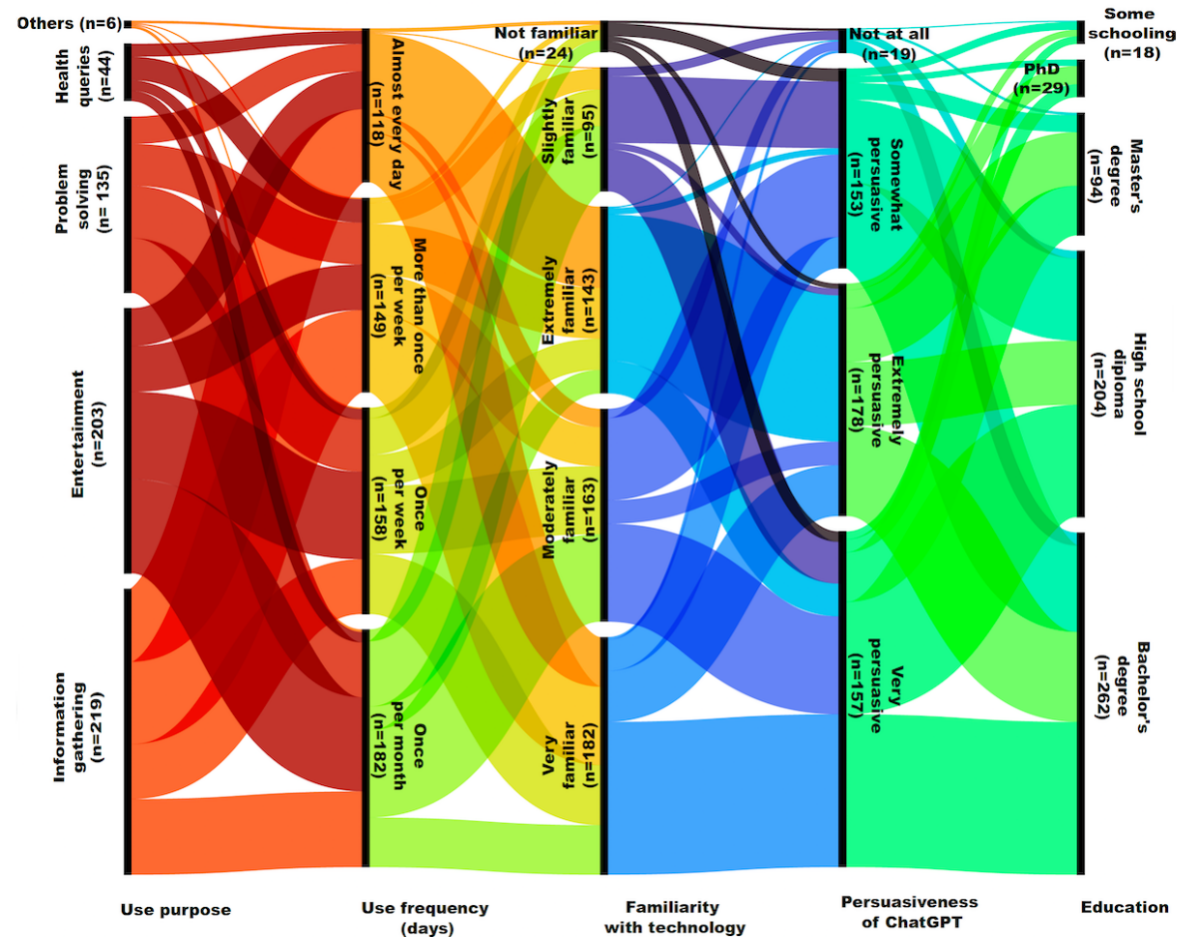
Illustration of the proportion of ChatGPT use frequency, respondents’ purpose of using ChatGPT, their familiarity with the technology, their perception of ChatGPT’s persuasiveness, and their education level. ChatGPT: Chat Generative Pretrained Transformer.

Figure 2 illustrates the proportion of ChatGPT use frequency, respondents’ purpose of using ChatGPT, their familiarity with the technology, their perception of ChatGPT’s persuasiveness, and their education level.

Our study investigated the relationships between performance expectancy, risk-reward appraisal, decision-making, and the intent to use ChatGPT for self-diagnosis. The *R*^2^ values indicated that our model can explain 52.4% of the variance in decision-making and 38.1% in the intent to use ChatGPT for self-diagnosis. When adjusting for the number of predictors in the model, the *R*^2^ values were 52.2% for decision-making and 37.9% for the intent to use ChatGPT for self-diagnosis, demonstrating satisfactory explanatory power.

Regarding reliability, the performance expectancy construct had a Cronbach α coefficient of .783, a rhoC of 0.860, and an AVE of 0.606. The decision-making construct exhibited a Cronbach α coefficient of .668, a rhoC of 0.858, and an AVE of 0.751. The rhoA values were similar to Cronbach α values for each construct, further supporting the reliability of the constructs. Moreover, the AVE values for all constructs exceeded the recommended threshold of 0.5, indicating adequate convergent validity.

**Table 2:** Descriptive statistics of study variables.

Question	Mean (SD)	Kurtosis
PE1^a^	3.24 (0.77)	0.694
PE2	3.22 (0.78)	0.285
PE3	3.20 (0.74)	0.290
PE4	3.24 (0.76)	0.403
DM1^b^	3.25 (0.78)	0.633
DM2	3.13 (0.81)	0.028
RRA^c^	3.20 (0.80)	0.371
IU^d^	3.09 (0.85)	–0.180

^a^PE: performance expectancy.

^b^DM: decision-making.

^c^RRA: risk-reward appraisal.

^d^IU: intent to use.

Our findings provide empirical support for all 3 hypotheses. We discovered that higher performance expectancy and positive risk-reward appraisals of ChatGPT are positively associated with improved decision-making outcomes among users. Additionally, enhanced decision-making processes involving ChatGPT positively impact users’ intention to use the technology for self-diagnosis. The results, including standardized β coefficients, SDs, *t* values, and 95% CIs, are presented in Table 3.

PLS-SEM analysis results elucidated the significant associations among the study variables. The direct effects indicated a significant positive association between performance expectancy and decision-making (β=.547, *t*=14.715) and between risk-reward appraisal and decision-making (β=.245, *t*=5.850). Moreover, the analysis identified a noteworthy positive relationship between decision-making and the intent to use (β=.565, *t*=16.928). Concerning indirect effects, the findings revealed that performance expectancy significantly influences the intent to use, mediated by decision-making (β=.309, *t*=10.911). Likewise, risk-reward appraisal demonstrated a meaningful positive impact on the intent to use via decision-making (β=.138, *t*=5.191).

The “total effects of study variables” section of Table 3 provides a comprehensive understanding of the overall influence of each variable on the others. Performance expectancy significantly affected decision-making (β=.547) and the intent to use (β=.309). In contrast, risk-reward appraisal substantially impacted decision-making (β=.245) and, indirectly, the intent to use (β=.138). In summary, PLS-SEM analysis offers crucial insights into the interrelationships among study variables, underscoring the salience of performance expectancy, risk-reward appraisal, and decision-making in shaping the intent to use.

**Table 3:** Standardized direct, indirect, and total effects.

Effects		β (SD)	*t* value	95% CI	
**Direct effects of study variables**					
	Performance expectancy à decision-making	.547 (.037)	14.715	0.474-0.620	
	Risk-reward appraisal à decision-making	.245 (.042)	5.850	0.161-0.325	
	Decision-making à intent to use	.565 (.033)	16.928	0.498-0.628	
**Indirect effects of study variables**					
	Performance expectancy à decision-making à intent to use	.309 (.028)	10.911	0.255-0.366	
	Risk-reward appraisal à decision-making à intent to use	.138 (.026)	5.191	0.087-0.191	
**Total effects of study variables**					
	Performance expectancy à decision-making	.547 (.037)	N/A^a^	0.474-0.620	
	Performance expectancy à intent to use	.309 (.028)	N/A	0.255-0.366	
	Risk-reward appraisal à decision-making	.245 (.042)	N/A	0.161-0.325	
	Decision-making à intent to use	.138 (.027)	N/A	0.087-0.191	

^a^N/A: not applicable.

## Discussion

### Principal Findings

We acknowledge that conversational AI systems, such as ChatGPT, can be crucial in health care by providing numerous possibilities to elevate patient care, optimize medical workflows, and augment the overall health care experience. In this study, we highlighted specific obstacles that must be tackled for secure and efficient implementation of ChatGPT. Although our study is the first to investigate the effects of perceived ChatGPT effectiveness and risk-reward appraisal on decision-making, its validity is supported by numerous studies examining the relationships among performance expectancy, technology acceptance, usage, risk-reward appraisal, and decision-making in other domains.

### Contributions of This Study

The findings of our study contribute to the growing body of the literature on AI chatbots in health care and their potential applications, particularly in the context of self-diagnosis. In recent years, research has increasingly focused on developing and evaluating AI chatbots for various health care purposes [5,36]. However, our study is unique in that it specifically examines the factors influencing users’ intentions to use ChatGPT, an AI chatbot not designed for health care purposes, for self-diagnosis. This novel focus allows for a deeper understanding of users’ perceptions and behaviors in the context of AI chatbots and self-diagnosis, which can be crucial for ensuring the safe and responsible integration of such technologies into health care.

Our research builds on earlier studies investigating the factors affecting the adoption of AI chatbots in health care [37-40]. Although these studies have provided valuable insights into the factors driving the adoption of AI chatbots, our study extends this knowledge by examining performance expectancy, risk-reward appraisal, and decision-making processes as key determinants of users’ intentions to use ChatGPT for self-diagnosis. This nuanced analysis can help inform the design and implementation of AI chatbots in health care and also help develop policies and interventions to mitigate the potential risks of using such technologies for self-diagnosis.

By focusing on ChatGPT, our study contributes to the broader conversation on AI chatbots’ ethical and societal implications in health care. The increasing popularity of AI chatbots, such as ChatGPT, for self-diagnosis raises important questions about the responsibilities of AI developers, health care providers, and policy makers in ensuring such technologies’ safe and responsible use. Our findings highlight the need for a collaborative, interdisciplinary approach to addressing these challenges, involving stakeholders from various sectors, including AI development, health care, policy, and ethics.

### Implications

The implications of our findings from a policy and pragmatic standpoint suggest a need for proactive preparation and policy alteration concerning the use of ChatGPT for self-diagnosis in health care.

Human behavior has consistently demonstrated a tendency to repurpose technology for purposes beyond its original design, even when aware of the potential risks or drawbacks. In the context of our study, people are inclined to use ChatGPT, a technology not specifically designed for health care applications, for self-diagnosis, as they perceive it to be useful and easy to use. Similarly, as evidenced by our prior study on internet use and mental health, people often turn to online sources for self-diagnosis and health information, despite the potential negative impact on mental health [41]. The reliance on these sources can be attributed to “curiosity gap” theory [42], which suggests that individuals are motivated to seek information to reduce uncertainty, even when the information may not be entirely accurate or reliable. This drive for information, combined with the convenience and accessibility of technology, may result in people using tools like ChatGPT or the internet for self-diagnosis, despite their inherent limitations.

In both cases, people’s behavior can be understood by observing the balance between perceived benefits, ease of use, and potential risks. The desire to reduce uncertainty and the convenience of technology may outweigh the awareness of potential drawbacks or misuse. This highlights the need to develop and regulate technologies like ChatGPT and online health information sources to meet health care applications’ unique requirements and ethical considerations, ensuring that they are user-friendly and trustworthy and minimize negative impacts on users’ health and well-being.

First, policy makers and health care stakeholders should collaborate to establish guidelines and ethical standards for using ChatGPT in health care settings [43]. These guidelines should consider the potential risks, benefits, and limitations of using AI-powered chatbots in health care, such as patient privacy, the health care applications’ unique requirements, and the ethical considerations researchers should focus on, to enhance the performance, safety, and accuracy of ChatGPT for health care applications.

Second, by tailoring the chatbot to address medical inquiries and concerns better, users can receive more reliable and valuable information to inform their decision-making processes. In addition, incorporating evidence-based medicine, reliable sources, and expert opinions into the chatbot’s knowledge base can further improve its credibility and usefulness in the health care [44]. To extend this implication, an integrated diagnostics mechanism could be developed to enhance ChatGPT’s ability to assist users with self-diagnosis. This mechanism would involve combining various diagnostic tools and techniques, such as symptom checkers, medical history analysis, and even integration with wearable health-monitoring devices, to gather real-time data. ChatGPT could then analyze the information provided by these sources to generate more accurate and personalized assessments of the user’s health condition.

Third, educating and informing users about the appropriate use of ChatGPT for self-diagnosis and its limitations are essential. Public health campaigns and educational materials should emphasize the importance of consulting health care professionals for accurate diagnosis and treatment, while highlighting the potential benefits of using chatbots as an adjunct tool for health information and decision-making support. A feedback mechanism could be proposed to ensure shared understanding and improve user awareness. This mechanism would involve users providing feedback on their experience with ChatGPT, including the accuracy and relevance of the information received and any concerns or misconceptions they may have encountered. Health care professionals could also be involved in this process, sharing their perspectives on the chatbot’s performance and suggesting improvements to enhance its reliability and user-friendliness. The feedback collected would then be used to refine ChatGPT’s algorithms, knowledge base, and user interface, ensuring it remains current with the latest medical knowledge and best practices. This iterative process would foster continuous improvement of the chatbot’s performance and promote greater awareness and understanding among users about the appropriate use of ChatGPT and its limitations in the context of self-diagnosis. Additionally, educational resources, such as tutorials and guidelines, could supplement the feedback mechanism to guide users in interacting with ChatGPT effectively and responsibly. By implementing a feedback mechanism and providing educational support, users can better perceive ChatGPT’s capabilities and limitations, ultimately promoting responsible and effective use of AI chatbots in health care settings.

Lastly, continuous monitoring and evaluation of ChatGPT’s use in health care should be conducted to assess its impact on health care outcomes and decision-making. This will enable policy makers and health care providers to make informed decisions about the potential benefits, risks, and practical applications of ChatGPT in health care settings.

### Limitations

Our study has limitations that warrant consideration. First, we did not control for potential confounding factors, such as age, medical condition, health literacy, previous experience with comparable technologies, or demographic characteristics, which might significantly influence users’ intentions to use ChatGPT for self-diagnosis. The results among younger and healthier populations could differ substantially from those among older populations with existing medical conditions. Younger individuals may be more inclined to use AI chatbots due to their familiarity with technology. In comparison, older individuals or those with medical conditions may seek additional reassurance or support for managing their health concerns.

Second, the cross-sectional survey design constrained our capacity to examine the evolving nature of users’ interactions with AI chatbots. Moreover, relying on self-reported measurements may introduce various biases, including social desirability, recall, or imprecise reporting. Self-report measures obtained through surveys inherently capture users’ perceptions rather than objective reality. Although the participants’ subjective experiences can provide valuable insights, there may be discrepancies between these perceptions and the actual situation. Furthermore, the cross-sectional design of the study limited our ability to draw causal inferences over time. Future research could use a triangulation approach to mitigate these limitations, incorporating objective measures and longitudinal data collection to provide a more comprehensive understanding of the phenomenon under investigation. Lastly, focusing on ChatGPT, which is not specifically intended for health care applications, may limit the applicability of the findings to other AI chatbots explicitly designed for health care purposes.

To address these limitations, future research should consider using longitudinal data, stratifying the sample by age group and medical condition, and accounting for potential confounding factors, such as participants’ familiarity with AI technology, prior experiences with chatbots, and demographic information. Various methodologies could provide additional insights, including monitoring chatbot usage and conducting qualitative interviews to assess trust and user behavior. Enhancing the data collection frequency and guaranteeing participant anonymity may also help reduce biases. By addressing these constraints, future research can contribute to a more comprehensive understanding of AI chatbot adoption in health care settings and enable more targeted interventions to optimize patient care and outcomes across diverse populations and health statuses.

### Conclusion

In conclusion, our research investigated the factors influencing users’ intentions to use ChatGPT for self-diagnosis, a purpose for which the technology is not specifically designed. The study aimed to explore the implications of these factors for the safe and effective integration of AI chatbots in health care settings. By examining performance expectancy, risk-reward appraisal, and decision-making processes, our findings contribute to the growing body of the literature on AI chatbots in health care and provide insights into AI chatbot adoption in health care contexts.

The clinical message of this study is to emphasize the importance of ongoing collaboration among AI developers, health care providers, and policy makers in ensuring the safe and responsible use of AI chatbots in health care. Addressing users’ expectations, risk-reward appraisal, and decision-making processes can help develop AI chatbots tailored to human needs and preferences, providing consumers with reliable and verified sources for health-related information. This approach can not only enhance health care accessibility but also improve health literacy and awareness among the public.

As the field of AI chatbots in health care continues to evolve, future research should further investigate the long-term effects of using AI chatbots for self-diagnosis and explore the potential integration of AI chatbots with other digital health interventions to optimize patient care and outcomes. In doing so, we can better understand the implications of AI chatbot usage in health care settings and ensure that these technologies are designed and implemented to safeguard users’ well-being and support positive health outcomes.

## Abbreviations

AI: artificial intelligence
AVE: average variance extracted
ChatGPT: Chat Generative Pretrained Transformer
e-commerce: electronic commerce
PLS-SEM: partial least squares structural equation modeling
PMT: protection motivation theory
TAM: technology acceptance model
UTAUT: unified theory of acceptance and use of technology
